# Linoleic acid and stearic acid are biosynthetic precursors of (7*Z*,10*Z*)-7,10-hexadecadienal, the major component of the sex pheromone of *Chilecomadia valdiviana* (Lepidoptera: Cossidae)

**DOI:** 10.1371/journal.pone.0215769

**Published:** 2019-04-23

**Authors:** Heidy Herrera, Wilson Barros-Parada, Jan Bergmann

**Affiliations:** 1 Instituto de Química, Facultad de Ciencias, Pontificia Universidad Católica de Valparaíso, Valparaíso, Chile; 2 Núcleo de Química y Bioquímica, Facultad de Estudios Interdisciplinarios, Universidad Mayor, Santiago, Chile; 3 Escuela de Agronomía, Facultad de Ciencias Agronómicas y de los Alimentos, Pontificia Universidad Católica de Valparaíso, Quillota, Chile; USDA Agricultural Research Service, UNITED STATES

## Abstract

The main pheromone compound of *Chilecomadia valdiviana* (Lepidoptera: Cossidae) has been recently identified as (7*Z*,10*Z*)-7,10-hexadecadienal. The biosynthesis of this pheromone compound showing attributes of both Type I and Type II lepidopteran pheromones was studied by the topical application of isotope-labeled fatty acids to the pheromone gland and subsequent analysis of the gland contents (pheromone compounds and fatty acyl compounds) by gas chromatography-mass spectrometry. The deuterium label of D_11_-linoleic acid was incorporated into the pheromone compound and its putative acyl precursor (7*Z*,10*Z*)-7,10-hexadecadienoate, demonstrating that the pheromone compound is biosynthesized from linoleic acid by chain-shortening and further functional group transformation. Furthermore, the deuterium label of D_3_-stearic acid was also incorporated into the pheromone compound, which indicates that the pheromone can be synthesized *de novo* by *C*. *valdiviana*, as is the case for Type I lepidopteran pheromone compounds.

## Introduction

The sex pheromones of about 600 species of Lepidoptera have been identified [1,2] and according to their structure, they are often classified as “Type I” or “Type II” pheromones. The common feature of Type I pheromones, produced by the majority of species, is an unbranched chain of 10–18 carbon atoms possessing a terminal oxygenated functional group (alcohol, aldehyde, or acetate) and 0–3 double bonds along the chain, while Type II pheromones, produced by a number of species in the families Arctiidae, Geometridae, Lymantriidae, and Noctuidae, are straight-chain, polyunsaturated hydrocarbons and their epoxy derivatives [1,3].

There is ample evidence that the biosynthetic pathways for the production of the two types of pheromones are quite different. The biosynthesis of Type I compounds takes place in the pheromone gland, where saturated fatty acid precursors (most often hexadecanoic (palmitic) or octadecanoic (stearic) acid) are transformed by a series of desaturation, chain shortening, and/or chain elongation steps to produce unsaturated fatty acid precursors of a defined chain length and with a certain number of double bonds with specific positions and geometries. Reduction of these acyl precursors, eventually followed by oxidation or esterification, finally produces the pheromone alcohols, aldehydes, and acetates, respectively [4].

In contrast, the polyunsaturated hydrocarbons representative of Type II pheromone compounds are derived from dietary (9*Z*,12*Z*)-9,12-octadecadienoic (linoleic) or (9*Z*,12*Z*,15*Z*)-octadecatrienoic (linolenic) acid precursors, which are subjected to chain elongation reactions followed by reductive decarboxylation in oenocyte cells. In some cases, a further dehydrogenation reaction may take place [5]. The resulting hydrocarbons are then transported to the pheromone gland; from where they are either released unchanged into the environment, or may suffer epoxidation before they are finally released [3,4].

Most species produce exclusively either Type I or Type II compounds, but there are examples of species producing compounds of both types [6–14] and there is evidence that in these species two distinct biosynthetic pathways exist, which independently produce the compounds of either type [15].

**Fig 1:**
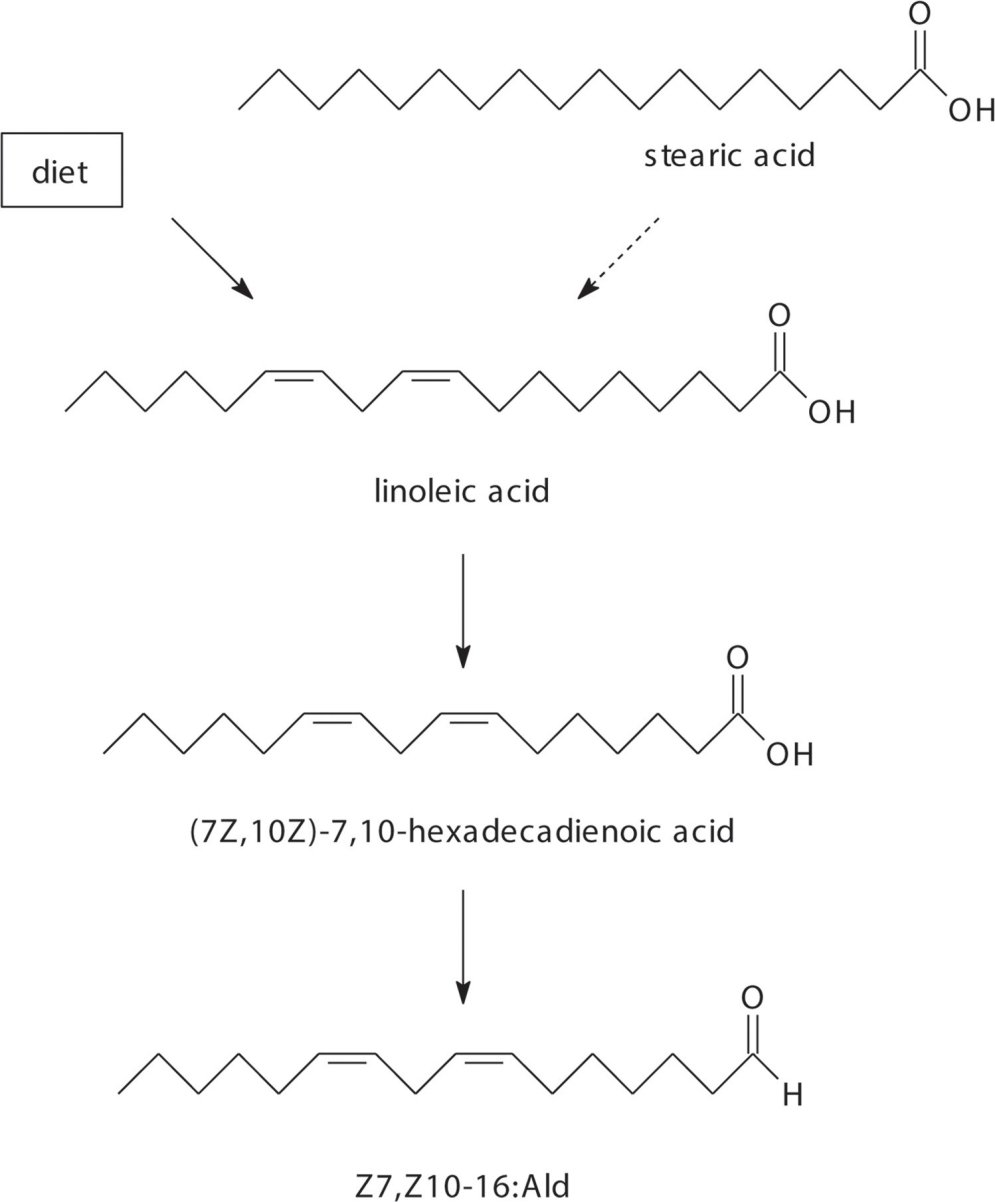
Proposed biosynthetic pathway for (7*Z*,10*Z*)-7,10-hexadecadienal from linoleic acid. The linoleic acid may be of dietary origin or may be synthesized from stearic acid (dashed arrow).

Particularly interesting in this context are pheromone compounds which according to their structure would be classified as Type I compounds, but which seem to be derived from linoleic or linolenic acids, which is typical for Type II compounds [16]. Examples for these compounds are (9*Z*,12*Z*)-9,12-octadecadienal (Z9,Z12-18:Ald) and (9*Z*,12*Z*,15*Z*)-9,12,15-octadecatrienal (Z9,Z12,Z15-18:Ald) identified from the Noctuidae *Achaea janata* [17], as well as from the Arctiidae *Amsacta albistriga* [17], *Diacrisia obliqua* [17], *Estigmene acrea* [18], and *Hyphantria cunea* [19]. These compounds are expected to be derived from linoleic and linolenic acid precursors, respectively, because they maintain the pattern of homoconjugated, (*Z*)-configured doubled bonds in the 9,12 or 9,12,15 positions present in the presumed precursors. Another example is (7*Z*,10*Z*)-7,10-hexadecadienal (Z7,Z10-16:Ald), recently identified as the main pheromone component of the Cossidae *Chilecomadia valdiviana* [20]. For this compound, chain-shortening of linoleic acid via β-oxidation, followed by functional group transformation is a plausible biosynthetic pathway (Fig 1). However, to date there is only limited evidence for the seemingly obvious biosynthesis of these compounds.

Thus, the objective of this study was to study the conversion of isotope-labeled putative fatty acid precursors in the pheromone gland of *C*. *valdiviana*, in order to gain more insight in the biosynthesis of Z7,Z10-16:Ald.

## Materials and methods

### Chemicals

14,14,15,15,16,16,17,17,18,18,18-D_11_-linoleic acid was purchased from Cayman Chemical (Ann Arbor, MI, USA). (*Z*)-9-Octadecenoic (oleic) acid uniformly labeled at each position with a ^13^C atom (^13^C-UL-oleic acid) was a gift from Prof. Christer Löfstedt (University of Lund, Sweden), who purchased it from Larodan (Solna, Sweden). 16,16,16-D_3_-palmitic acid and 18,18,18-D_3_-stearic acid were purchased from Sigma-Aldrich (St. Louis, MO, USA). A 37 compound fatty acid methyl ester (FAME) mix from Supelco was used as reference (Merck, Darmstadt, Germany). Methyl (7*Z*,10*Z*)-7,10-hexadecadienoate (Z7,Z10-16:COOMe) and methyl (*Z*)-7-hexadecenoate (Z7-16:COOMe) were obtained by oxidation with Jones reagent [21] and subsequent methylation by BF_3_-methanol [22] of (7*Z*,10*Z*)-7,10-hexadecadien-1-ol and (*Z*)-7-hexadecen-1-ol, respectively, which were available in our laboratory from earlier work [20].

### Insects

*Chilecomadia valdiviana* is not an endangered or protected species. A permit from the Institutional Animal Care and Use Committee was not required for work with this invertebrate species. Insects were collected as pupae from infested apple tree logs in the localities of Colbún and San Javier (Maule Region, central Chile) with permission from the orchard managers. After transport to the laboratory, pupae were separated according to their sex and maintained individually in 50 mL polystyrene containers under controlled conditions (20°C, 60–70% r.h., 12 L:12D) until the emergence of adults.

### Application of labeled precursors

For the application of labeled fatty acids, 1 d-old virgin females were used. To facilitate handling, insects were sedated by maintaining them at 5°C for 15 min prior to application of precursor solutions. A solution of 40 μμg of the respective fatty acid in 1 μL dimethyl sulfoxide (DMSO) (anhydrous, Sigma-Aldrich) was applied topically to the pheromone gland (located in the terminal segments of the abdomen) during the 4^th^ hour of the scotophase. For control samples, DMSO (1 μL) was applied. The following fatty acids were applied individually (number of repetitions in parenthesis): D_11_-linoleic acid (6), ^13^C-UL-oleic acid (10), D_3_-stearic acid (8), and D_3_-palmitic acid (5). After 4 h, the insects were freeze-killed at -20°C, and the pheromone gland was excised and immersed in 50 μL of hexane (SupraSolv, Merck, Darmstadt, Germany). After 10 min, the hexane was carefully removed and transferred to another vial, and the sample was either analyzed immediately or stored at -20°C until analysis. To the remaining tissue was added 50 μL of chloroform (p.a., Merck, Darmstadt, Germany). After 2 h, the solvent was separated, 50 μL of 20% boron trifluoride-methanol was added to the extract and the mixture was maintained at 60°C for 2 h [22]. After cooling to room temperature, water (100 μL) and hexane (100 μL) were added. The organic phase was carefully separated, dried (Na_2_SO_4_), and analyzed by gas chromatography coupled with mass spectrometry (GC/MS) as described below. The extractions were performed with single glands, and the extracts were analyzed individually.

The titer of labeled and unlabeled pheromone compounds was calculated based on the area under the peaks of the molecular ions (M^+^) in the respective mass chromatograms (*m/z* 236, 239, and 247 for the unlabeled compound, the D_3_-labeled compound, and the D_11_-labeled compound, respectively), referring to a calibration curve made with authentic Z7,Z10-16:Ald. The data for ratios of the titers of labeled/unlabeled pheromone were analyzed by Mann-Whitney *U* test, to detect differences between the values and from 0, using R 3.2.3 GUI 1.66 Mavericks build (R Core team).

### Derivatization with dimethyl disulfide

Dimethyl disulfide (DMDS) (50 μL) and 50 μL of a 5% solution of iodine in diethyl ether were added to a methylated chloroform gland extract (ca. 50 μL) [23]. The reaction mixture was kept at 50°C overnight. After addition of a drop of an aqueous solution of 10% sodium thiosulfate, hexane (200 μL) was added. The hexane solution was carefully separated, concentrated to ca. 20μL, and analyzed by GC/MS using the RTX-5 column as described below.

### Chemical analyses

GC/MS was carried out using a Shimadzu GCMS-QP2010 Ultra combination (Shimadzu, Kyoto, Japan) using either a fused silica RTX-5 capillary column (30 m × 0.25 mm id, 0.25 mm film, Restek, Bellefonte, PA, USA) or a fused silica SP2380 capillary column (30 m × 0.25 mm id, 0.2 μm film, Varian Inc., Lake Forest, CA, USA). For the RTX-5 column, the oven was programmed from 50°C for 2 min and then at 8°C min^-1^ to 270°C, and for the SP2380 column from 50°C for 2 min, and then at 8°C min^-1^ to 230°C. The GC was operated in split/splitless mode (30 s sampling time) with an injector temperature of 200°C. The injection volume was 1 μL. Helium was used as the carrier gas at column flow rates of 1.22 mL min^-1^ (RTX-5) and 1.00 mL min^-1^ (SP2380). Electron impact mass spectra were acquired at 70 eV in full scan mode (*m/z* 35–500).

## Results

### Analysis of the fatty acid composition in the pheromone gland

Comparison of the retention times on the SP2380 stationary phase and the mass spectra of the FAME present in methylated chloroform extracts with those of the reference compounds, allowed the identification of 20 fatty acyl compounds in the pheromone gland (Table 1, S1 Fig). The most abundant compounds in the extract were the methyl esters of oleic, palmitic, and linoleic acids, followed by moderate amounts of the methyl esters of (*Z*)-9-hexadecenoic (palmitoleic), stearic, and linolenic acids. In addition to these esters derived from commonly found fatty acids, other methyl esters, including Z7,Z10-16:COOMe and Z7-16:COOMe, were present in minor or trace amounts. These last two compounds are expected to be direct precursors of the main pheromone compound Z7,Z10-16:Ald and of Z7-16:Ald, respectively, and their identity was further confirmed by matching retention times with those of the respective reference compounds on the RTX-5 stationary phase. Interpretation of the diagnostic fragments in the mass spectra of the compounds in the DMDS-derivatized chloroform gland extract confirmed the location of the double bonds of some of the monounsaturated compounds, and revealed additionally the presence of methyl 11-octadecenoate (S1 Table).

**Table 1:** Methyl esters of fatty acids identified in a methylated chloroform extract of the pheromone gland of *Chilecomadia valdiviana*.

N°	Compound	Relative amount (%)^a^
1	Methyl dodecanoate	0.08 ± 0.02
2	Methyl tetradecanoate	0.21 ± 0.07
3	Methyl hexadecanoate	26.9 ± 2.19
4	Methyl hexadecenoate^b^	0.03 ± 0.03
5	Methyl (*Z*)-7-hexadecenoate	0.39 ± 0.11
6	Methyl (*Z*)-9-hexadecenoate	3.36 ± 1.22
7	Methyl hexadecenoate^b^	0.05 ± 0.04
8	Methyl (7*Z*,10*Z*)-7,10-hexadecadienoate	0.23 ± 0.08
9	Methyl octadecanoate	3.58 ± 1.73
10	Methyl (*Z*)-9-octadecenoate	50.0 ± 4.35
11	Methyl octadecadienoate^b^	0.71 ± 0.22
12	Methyl (9*Z*,12*Z*)-9,12-octadecadienoate	11.8 ± 0.60
13	Methyl eicosanoate	0.29 ± 0.15
14	Methyl (9*Z*,12*Z*,15*Z*)-9,12,15-octadecatrienoate	1.35 ± 0.49
15	Methyl (*Z*)-11-eicosenoate	0.08 ± 0.04
16	Methyl (11*Z*,14*Z*)-11,14-eicosadienoate	0.03 ± 0.02
17	Methyl docosanoate	0.15 ± 0.14
18	Methyl (5*Z*,8*Z*,11*Z*,14*Z*)-5,8,11,14-eicosatetraenoate	0.04 ± 0.06
19	Methyl (5*Z*,8*Z*,11*Z*,14*Z*,17*Z*)-5,8,11,14,17-eicosapentaenoate	0.70 ± 0.28
20	Methyl tetracosanoate	0.03 ± 0.01

^a^Mean ± SD (*N* = 4)

^b^Tentative identification; position and geometry of double bond(s) unknown

### Conversion of labeled fatty acids

#### D_11_-linoleic acid

**Fig 2:**
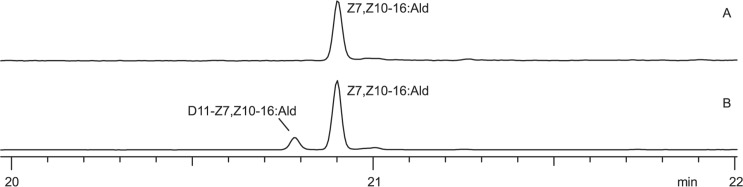
After topical application of D_11_-linoleic acid, a new peak eluting several seconds earlier than the pheromone compound appears. Gas chromatograms of hexane extracts of (A) a pheromone gland treated with DMSO only and (B) a pheromone gland treated with a solution of D_11_-linoleic acid in DMSO. Z7,Z10-16:Ald = (7*Z*,10*Z*)-7,10-hexadecadienal.

**Fig 3:**
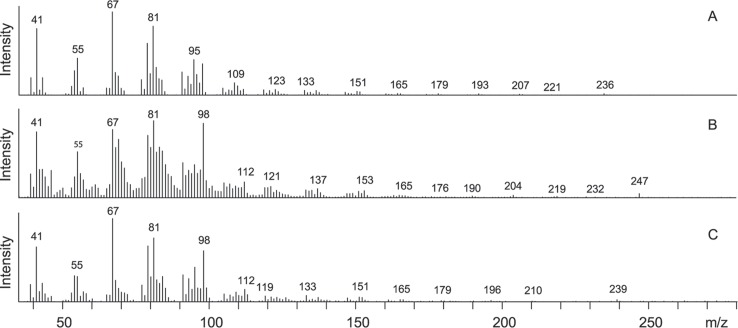
Mass spectra show the incorporation of the isotope label of topically applied D_11_-linoleic acid and D_3_-stearic acid into the pheromone compound. Mass spectra of (A) natural (7*Z*,10*Z*)-7,10-hexadecadienal, (B) D_11_-(7*Z*,10*Z*)-7,10-hexadecadienal from glands treated with D_11_-linoleic acid, (C) D_3_-(7*Z*,10*Z*)-7,10-hexadecadienal from glands treated with D_3_-stearic acid.

The GC/MS analysis of hexane extracts of pheromone glands treated with D_11_-linoleic acid revealed the production of deuterium-labeled Z7,Z10-16:Ald in 5 of 6 treated individuals (Table 2). Due to the extensive deuterium labeling, the exogenous labeled aldehyde (D_11_-Z7,Z10-16:Ald) elutes several seconds earlier than the endogenous unlabeled aldehyde (Z7,Z10-16:Ald) (Fig 2). Compared to the respective fragment ions in the mass spectrum of the unlabeled aldehyde (Fig 3A), the higher mass region of the mass spectrum of the labeled aldehyde exhibits fragment ions at *m/z* 176 (165 + 11), 190 (179 + 11), and 204 (193 + 11), and a molecular ion at *m/z* 247 (236 + 11) (Fig 3B), indicating the presence of 11 deuterium atoms in these fragments. Additionally, abundant fragment ions were observed in the lower mass region of D_11_-Z7,Z10-16:Ald, corresponding to fragments containing different numbers of deuterium atoms.

**Table 2:** Titers of unlabeled and labeled Z7,Z10-16:Ald in pheromone glands of *Chilecomadia valdiviana* females, which were treated with isotope-labeled fatty acids.

	Unlabeled pheromone		Labeled pheromone		Ratio [%]^c^
Treatment	Titer [ng/gland]^a^	Range [ng/gland]	Titer [ng/gland]^b^	Range [ng/gland]	
D_11_-linoleic acid	7.04 ± 4.65	0.68–13.5	0.82 ± 0.66	0.12–1.77	14.0 ± 8.4
D_3_-stearic acid	41.0 ± 32.2	15.2–77.1	0.67 ± 0.77	0.20–1.55	1.38 ± 0.61
D_3_-palmitic acid	31.1 ± 36.0	4.79–94.5	n.d.	—	—
^13^C-UL-oleic acid	16.5 ± 21.7	1.28–53.8	n.d.	—	—
control	2.72 ± 2.73	1.04–18.8	n.d.	—	—

^a^Mean ± SD; *N* = 5 for D_11_-linoleic acid, D_3_-palmitic acid, control; *N* = 7 for ^13^C-UL-oleic acid; *N* = 3 for D_3_-stearic acid

^b^Mean ± SD; *N* = 5 for D_11_-linoleic acid, *N* = 3 for D_3_-stearic acid, n.d. = not detected

^c^Values are different from each other and from 0 according to Mann-Whitney *U* test (P < 0.05)

**Fig 4:**
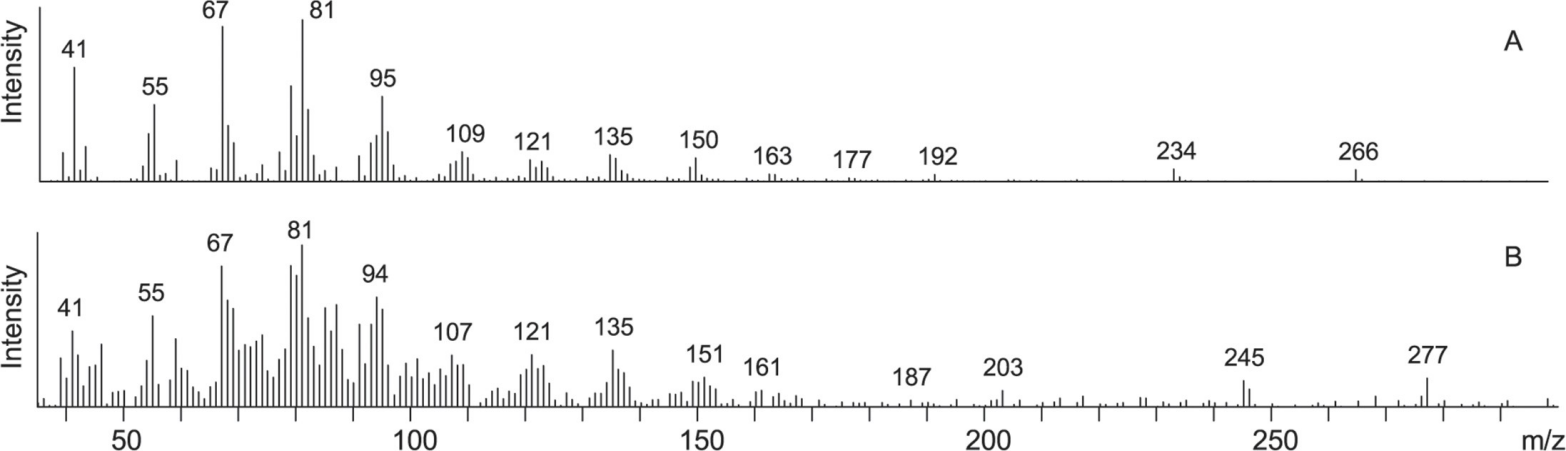
Linoleic acid is chain-shortened to (7*Z*,10*Z*)-7,10-hexadecadienoate in the pheromone gland. Mass spectra of (A) unlabeled methyl (7*Z*,10*Z*)-7,10-hexadecadienoate from methylated pheromone gland extracts, (B) methyl D_11_-(7*Z*,10*Z*)-7,10-hexadecadienoate from pheromone glands treated with D_11_-linoleic acid.

In a methylated chloroform extract of the pheromone gland treated with D_11_-linoleic acid, an additional peak eluting several seconds earlier than Z7,Z10-16:COOMe was observed as compared to control gland extracts (S2 Fig). This compound showed a mass spectrum consistent with D_11_-Z7,Z10-16:COOMe (Fig 4). The molecular ion was observed at *m/z* 277 (266 + 11), and the *m/z* values of the characteristic ions at 161, 203, and 245 indicated the presence of 11 deuterium atoms in these fragments. Again, abundant fragment ions were observed in the lower mass region of the labeled compound.

#### D_3_-stearic acid

**Fig 5:**
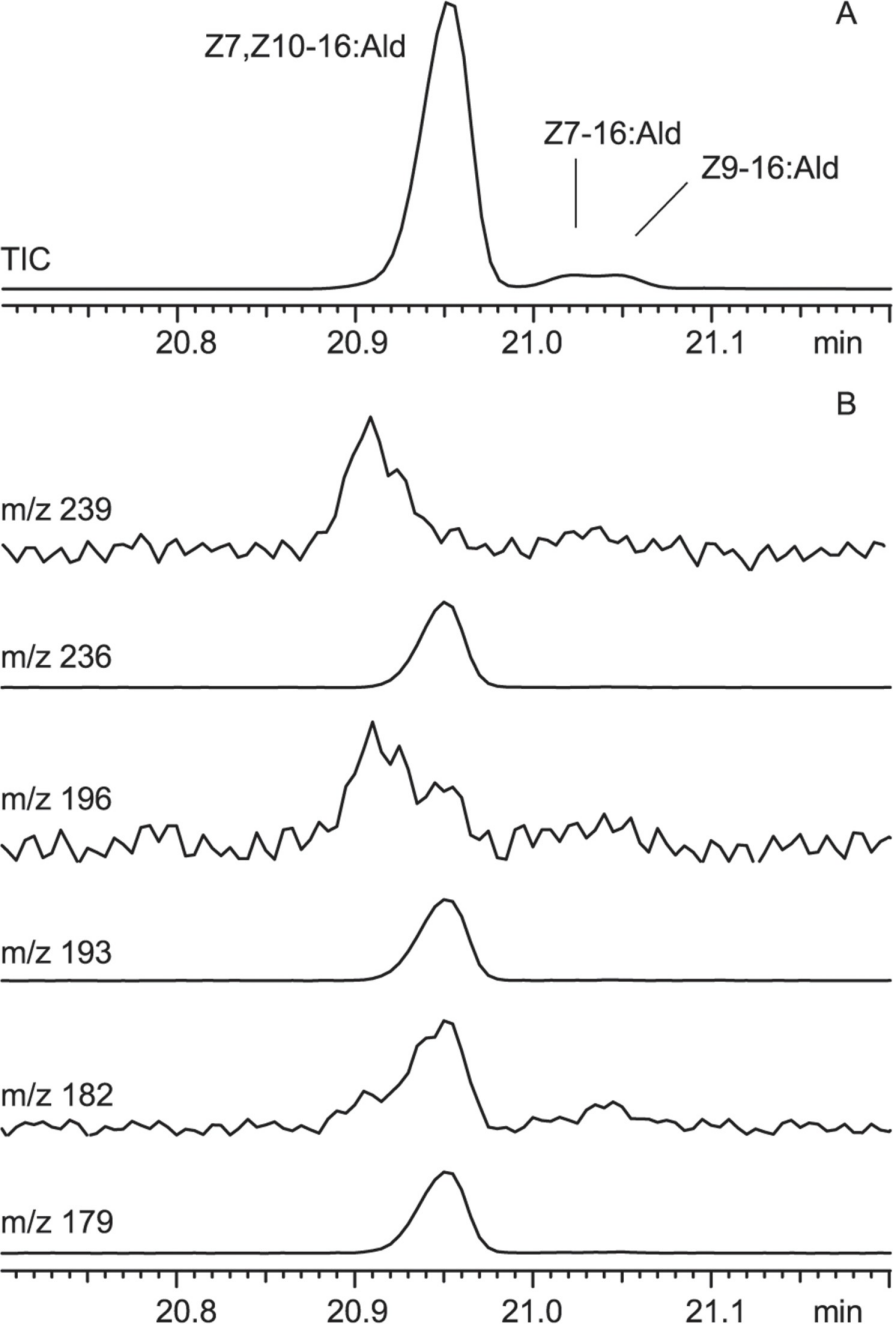
The isotope label of topically applied D_3_-stearic acid is incorporated into the pheromone component. (A) Total ion chromatogram (TIC) of a hexane extract of a pheromone gland treated with D_3_-stearic acid. Z7,Z10-16:Ald = (7*Z*,10*Z*)-7,10-hexadecadienal, Z7-16:Ald = (*Z*)-7-hexadecenal, Z9-16:Ald = (*Z*)-9-hexadecenal. (B) Mass chromatograms show the elution of the isotope-labeled pheromone compound (*m/z* 239, 196, and 182) slightly earlier than the non-labeled compound (*m/z* 236, 193, and 179).

In 3 of the 8 individuals treated with D_3_-stearic acid, we obtained evidence for the production of D_3_-Z7,Z10-16:Ald (Table 2). Fig 5 shows the total ion chromatograms and selected mass chromatograms of hexane extracts of a pheromone gland. The appearance of the molecular ion at *m/z* 239 (236 + 3) and of the fragment ions at *m/z* 182 (179 + 3) and 196 (193 + 3) in the mass spectrum suggested the incorporation of the label into the pheromone compound (Fig 3C). In addition to these ions, additional peaks and altered relative intensities of the peaks of the fragment ion clusters around *m/z* 55, 67, 81, 95, 109, and 123 were observed, further supporting the incorporation of deuterium atoms into the molecule.

#### Other isotope-labeled precursors

No labeled aldehyde was detected in extracts of glands treated with D_3_-palmitic acid (5 individuals) or ^13^C-UL-oleic acid (10 individuals).

## Discussion

The *in vivo* experiments carried out in this study confirmed the transformation of deuterium-labeled linoleic acid to deuterium-labeled Z7,Z10-16:Ald in the pheromone gland of *C*. *valdiviana* females. The first step in this transformation is the chain-shortening of linoleic acid by two carbons, as was evidenced by the appearance of deuterium-labeled Z7,Z10-16:COOMe among the fatty acyl compounds in the pheromone gland after treatment with D_11_-linoleic acid. This fatty acyl precursor is then further transformed to Z7,Z10-16:Ald, presumably by reduction to the corresponding primary alcohol and partial oxidation to the target aldehyde [24]. Another possibility is the direct reduction of the acyl precursor to the aldehyde [4]. We did not detect linoleyl alcohol, neither labeled nor unlabeled, in the extracts, but this may be accounted for by immediate oxidation of the alcohol to the aldehyde, which would prevent accumulation to detectable amounts in the gland.

The criteria generally used to differentiate Type I and Type II pheromones are the origin of precursors and site of biosynthesis–*de novo* from acetate in the pheromone gland (Type I) or from dietary linoleic/linolenic acid in oenocyte cells (Type II), respectively. Our results provide evidence that linoleic acid is subjected to chain-shortening in the pheromone gland. If the site of biosynthesis is the main criterion, then the fact that this transformation occurs in the pheromone gland would argue in favor of a Type I classification of Z7,Z10-16:Ald. It should be noted that *C*. *valdiviana* is from the Cossidae, from which only Type I compounds have been identified so far, while the species producing Z9,Z12-18:Ald and Z9,Z12,Z15-18:Ald belong to families known to produce Type II compounds (namely, Noctuidae and Arctiidae), and that for example *H*. *cunea* produces Z9,Z12-18:Ald and Z9,Z12,Z15-18:Ald together with “true” Type II compounds. It has to be kept in mind, however, that these categories do not exist as such in nature, and that the classification of a given compound depends on the criteria applied.

There are only two other examples where the biosynthesis of structurally similar compounds has been examined, and in both cases only functional group transformation, but no chain modification was involved. In particular, it was shown that *E*. *acrea* females incorporate the radioactivity of ^14^C-labeled linoleic and linolenic acids injected in pupae into the aldehyde fraction containing Z9,Z12-18:Ald and Z9,Z12,Z15-18:Ald, respectively [25]. Another study demonstrated that *H*. *cunea* is able to convert topically applied ^13^C-labeled linolenyl alcohol to Z9,Z12,Z15-18:Ald in the pheromone gland, but there was no evidence for conversion of ^13^C-labeled linolenic acid [26].

Other Type I pheromone compounds with a skipped diene system (i.e. possessing a methylene group between the double bonds) are relatively scarce in Lepidoptera. Examples include (4*E*,7*Z*)-4,7-tridecadienyl acetate from the Gelechiidae *Phthorimaea operculella* [27], (11*E*,14*E*)-11,14-octadecadienal from the Endromidae *Andraca bipunctata* [28], and 9,12-tetradecadienyl compounds identified from several species of Pyralidae and Noctuidae [2]. Although (4*E*,7*Z*)-4,7-tridecadienyl acetate was suggested to be derived from linoleic acid [29], the position and/or geometry of the double bonds of the latter two examples suggest that they are derived from saturated precursors. In fact, the biosynthesis of (9*Z*,12*E*)-9,12-tetradecadienyl acetate produced by *Cadra cuatella* and *Spodoptera exigua* was shown to start from palmitic acid [30].

At this point, the question was whether Z7,Z10-16:Ald is produced exclusively by transformation of (dietary) linoleic acid, or if females are also able to transform saturated precursors to the pheromone compound. The reproducible production of D_3_-Z7,Z10-16:Ald after topical application of D_3_-stearic acid to the gland indicates that stearic acid can be a precursor of the pheromone compound, which would also argue in favor of Z7,Z10-16:Ald being a Type I compound. A plausible pathway for this transformation is the production of oleic acid from stearic acid, followed by Δ12 desaturation to produce linoleic acid, which can then enter the biosynthetic pathway described above. In this case, one could expect the isotope label of externally applied ^13^C-UL-oleic acid to be carried through to the pheromone compound. However, we did not obtain evidence for the production of ^13^C-labeled Z7,Z10-16:Ald, Z7,Z10-16:COOMe, or linoleate in the respective experiments. A possible explanation is that the large amount of natural oleic acid dilutes the ^13^C-labeled oleic acid, which, as a consequence, is scarcely converted to Z7,Z10-16:Ald [26]. On the other hand, it is also possible that oleic acid is not a pheromone precursor at all and that the pathway involves the conversion of stearic acid to (*Z*)-12-octadecenaote, followed by a further desaturation step to produce linoleate. In any of these cases, a less efficient incorporation of the label as compared to linoleic acid, as observed, can be expected, because stearic acid is further upstream than linoleic acid.

It has long been assumed that linoleic acid is an essential nutrient that cannot be synthesized by animals, and it was not until the 1980s that the ability for *de novo* synthesis of linoleic acid was recognized in insects [31]. Several insect species from six orders [32–35], as well as various species of Collembola [36] are now known to be able to synthesize linoleic acid *de novo*, but this does not seem to be a universal feature in insects, and has not been reported so far for Lepidoptera.

## Conclusion

We have shown that linoleic acid is a direct precursor for the biosynthesis of Z7,Z10-16:Ald in *C*. *valdiviana*. We have not conclusively established the origin of linoleic acid used in pheromone biosynthesis, which might be either of dietary origin or be synthesized *de novo*, or a mixture of both. The chain-shortening of linoleic acid in the pheromone gland and the transformation of stearic acid to the pheromone compound suggests that the biosynthesis of Z7,Z10-16:Ald follows a pathway more similar to that for Type I lepidopteran pheromone compounds.

## Supporting information

S1 Fig Gas chromatogram of a methylated chloroform pheromone gland extract of *C. valdiviana*. Peak numbers correspond to compounds as listed in Table 1 in the main article.
(TIF)

S2 Fig Gas chromatogram of a methylated chloroform extract of the pheromone gland treated with D_11_-linoleic acid. Z7Z10-16:COOMe = methyl (7*Z*,10*Z*)-7,10-hexadecadienoate, 16:1 (c7) = methyl (*Z*)-7-hexadecenoate, 16:1 (c9) = methyl palmitoleate, 16:1 = methyl hexadecenoate (position and geometry of double bond unknown), 16:0 = methyl palmitate.
(TIF)

S1 Table Retention times and diagnostic fragments of DMDS-derivatives of monounsaturated fatty acid methyl esters identified from methylated pheromone gland extracts of *C. valdiviana*. (DOCX)

S1 Dataset Pheromone titers in glands of female *C. valdiviana*. (XLSX)
