# Supplementary figures and images for: Linoleic acid and stearic acid are biosynthetic precursors of (7Z,10Z)-7,10-hexadecadienal, the major component of the sex pheromone of Chilecomadia valdiviana (Lepidoptera: Cossidae)
Source: PLoS One. 2019 Apr 23;14(4):e0215769. doi: 10.1371/journal.pone.0215769 (PMC6478319; doi:10.1371/journal.pone.0215769)

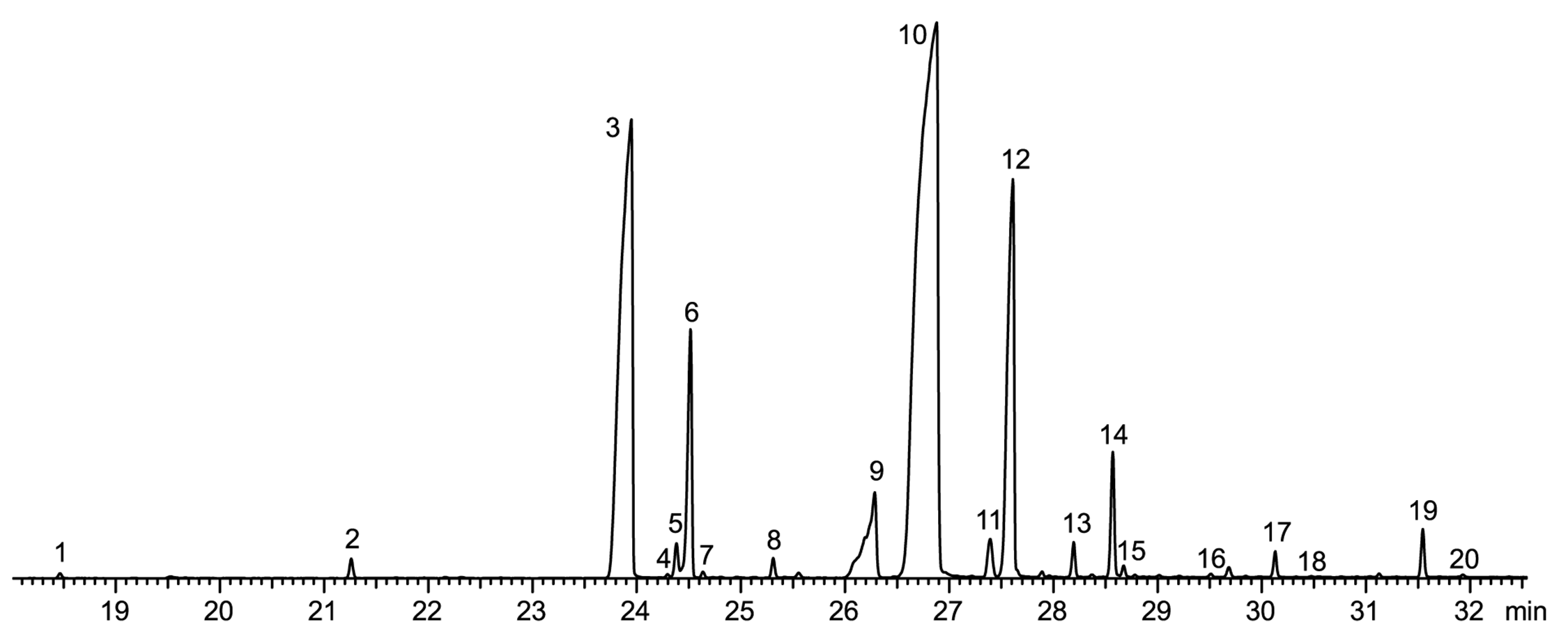

Supplement: S1 Fig — Peak numbers correspond to compounds as listed in Table 1 in the main article. (TIF) [file pone.0215769.s001.tif]

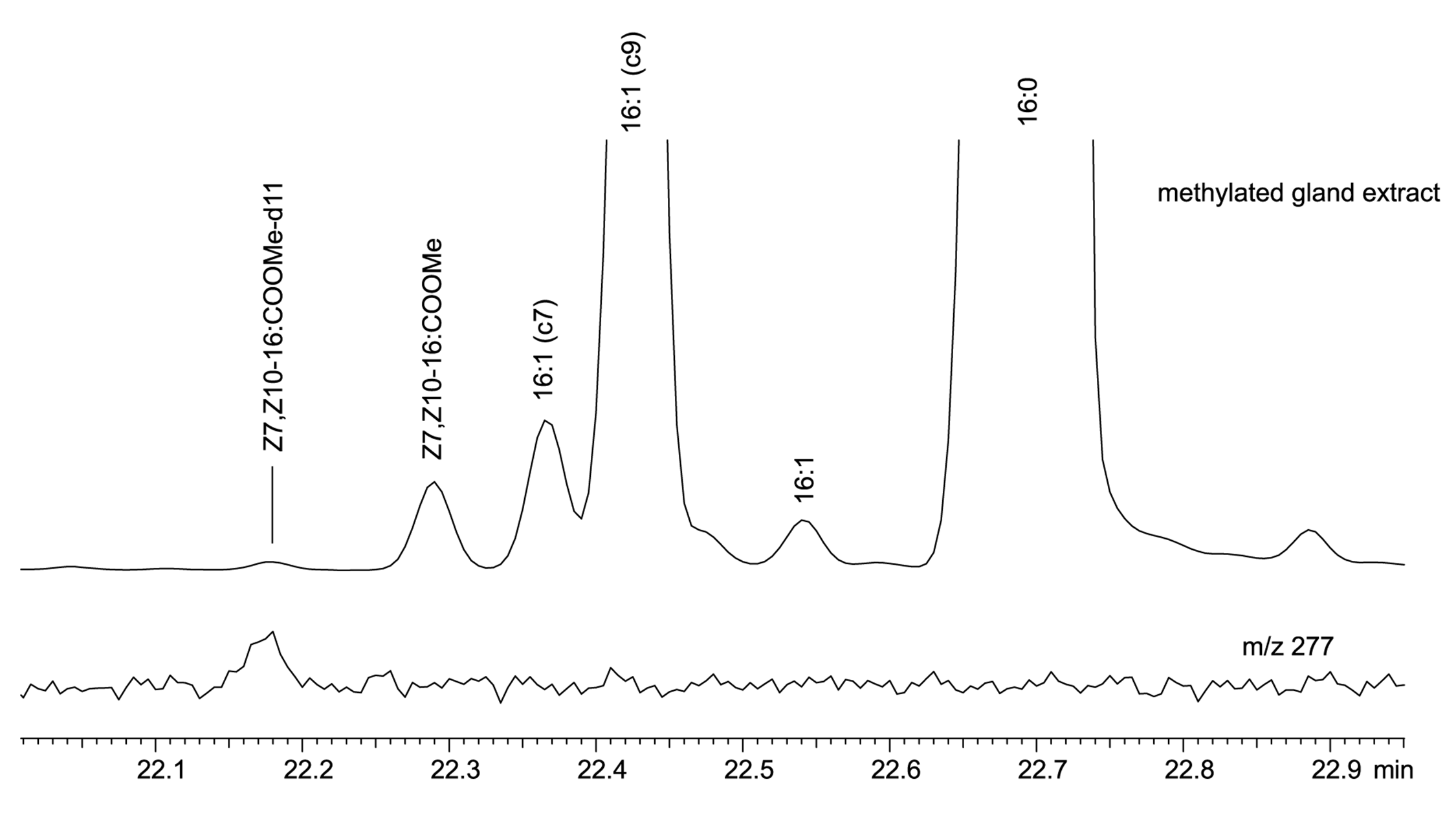

Supplement: S2 Fig — Z7Z10-16:COOMe = methyl (7Z,10Z)-7,10-hexadecadienoate, 16:1 (c7) = methyl (Z)-7-hexadecenoate, 16:1 (c9) = methyl palmitoleate, 16:1 = methyl hexadecenoate (position and geometry of double bond unknown), 16:0 = methyl palmitate. (TIF) [file pone.0215769.s002.tif]
