# Supplementary material for: Linoleic acid and stearic acid are biosynthetic precursors of (7Z,10Z)-7,10-hexadecadienal, the major component of the sex pheromone of Chilecomadia valdiviana (Lepidoptera: Cossidae)
Source: PLoS One. 2019 Apr 23;14(4):e0215769. doi: 10.1371/journal.pone.0215769 (PMC6478319; doi:10.1371/journal.pone.0215769)
Supplement: S1 Table — (DOCX) [file pone.0215769.s003.docx]

**S1 Table. Retention times and diagnostic fragments of DMDS-derivatives of monounsaturated fatty acid methyl esters identified from methylated pheromone gland extracts of *Chilecomadia valdiviana***

| Retention time (min) | Compound | Diagnostic fragments of DMDS adduct (*m/z*) |
| --- | --- | --- |
| 29.59 | Methyl (*Z*)-7-hexadecenoate | 173, 189, 362 |
| 29.63 | Methyl palmitoleate | 145, 217, 362 |
| 31.91 | Methyl oleate | 173, 217, 390 |
| 31.99 | Methyl 11-octadecenoate | 145, 245, 390 |
